# A case report of severe degenerative lumbar scoliosis associated with windswept lower limb deformity

**DOI:** 10.1186/s12893-020-00857-x

**Published:** 2020-09-03

**Authors:** Xi Yang, Qiang Zou, Yueming Song, Limin Liu, Chunguang Zhou

**Affiliations:** Department of Orthopedics Surgery and Orthopedics Research Institute, West China Hospital, Sichuan University, No. 37 GuoXue Road, Chengdu, Sichuan China

**Keywords:** Degenerative scoliosis, Windswept lower limb deformity, Leg length discrepancy, Pelvic obliquity, Rickets

## Abstract

**Background:**

The windswept lower limb deformity describes valgus deformity in one leg with varus deformity in the other. It is mostly seen in young children with metabolic bone diseases (such as rickets) and may lead to leg length discrepancy (LLD) and Degenerative scoliosis (DS) in older age. To the best of our knowledge, there was no report of the spinal surgery in patient with severe DS associated with windswept deformity. The objective of this study is to report the unique case of a 60-year-old woman with severe degenerative scoliosis (DS) associated with windswept deformity caused by rickets who underwent a posterior correction and fusion surgery in spine.

**Case presentation:**

The patient was diagnosed as rickets windswept lower limb deformity for 50 years but never went through routine treatment. Then, she performed lumbar scoliosis for more than 20 years and suffered from severe back pain for 4 years. After overall clinical evaluation and radiographic measures, we performed a posterior surgical correction and fusion from T9-L5. With this surgery, the main thoracolumbar curve Cobb angle corrected from 72.5° to 21.0°, the coronal balance from 0 cm to 2.0 cm while the sagittal vertical axis (SVA) from 1.5 cm to − 1.0 cm. At 2 years postoperative follow-up, her back pain has almost completely relieved with a satisfied fixation and bone fusion showed on CT scans. However, a coronal imbalance was found with C7-CSVLdistance equal to 4.0 cm. This coronal imbalance was highly correlated to the untreated LLD and pelvic obliquity, and should be improved by standing posture or shoe lifts.

**Conclusions:**

For such patient, the pure spinal correction and fusion surgery, in spite of lower limbs deformity, can achieve good relieve of back pain symptom, however may accompany by the complication of coronal imbalance due to the unimproved pelvic obliquity and LLD. However, longer follow-up is necessary to observe the long-term outcome of this patient’s postoperative coronal imbalance.

## Background

Degenerative scoliosis (DS) is a common condition in elderly patients [1]. The multiple factors associated with aging, including disc degeneration, facet joint arthrosis and vertebral rotatory olisthesis, have been recognized as main cause of DS [2–4]. Besides these, the leg length discrepancy (LLD) appearing during growth period has been considered as an initiating factor of DS in recent [5, 6]. The windswept lower limb deformity describes a kind of rare appearance of abnormal valgus deformity in one knee in association with varus deformity in the other [7]. It is mostly seen in young children with metabolic bone diseases, such as rickets, hypophosphatasia or renal osteodystrophy [8]. And the windswept deformity usually leads to the LLD in childhood that will finally result in the de novo lumbar scoliosis or degenerative scoliosis in adult age [9].

When the patients with lumbar scoliosis suffer from significant lower back pain and conservative therapy is ineffective, spinal fusion surgery is recommending. In such case, what’s role the windswept deformity plays in the spinal corrective surgery? Whether the lower limbs need correction subsequently? These are new issues that spinal surgeon need to face. Here, we present a unique case of an old woman with severe DS associated with windswept deformity caused by rickets who underwent a posterior correction and fusion surgery in spine. To the best of our knowledge, there was no published literature report of such kind of case.

## Case presentation

A 60-year-old short-statured woman experienced the lumbar scoliosis for more than 20 years, suffered from progressive lower back pain for 4 years (since from 2013). Besides, she also performed the lower limbs malformation for more than 50 years. She was diagnosed as rickets in her childhood but did not receive any regular effective treatment because of her previous poor family condition. Since her back pain initially occurred, she began receiving the oral administration NSAIDs, physical therapy, and facet joints injection treatment successively and repeatedly in our hospital. However, the symptom relief from above ways was temporary and limited. The painful gradually became intolerable (VAS = 8) that brought her back to our clinic found help through spine surgery. No leg pain, numbness, or claudication accompanied with her back pain all the time.

Physical examination showed her weight of 37 kg and height of 138 cm. The obvious scoliosis with curve convex to right side was found. Tenderness existed in her lumbar spinous process and paravertebral muscles. Both lower extremities, especially the right femur and left tibia, were bow appearance. Left leg is about 2.5 cm longer than the right one. The neurology function exam is normal. And bilateral hip and knee joint activity are almost normal.

**Fig. 1:**
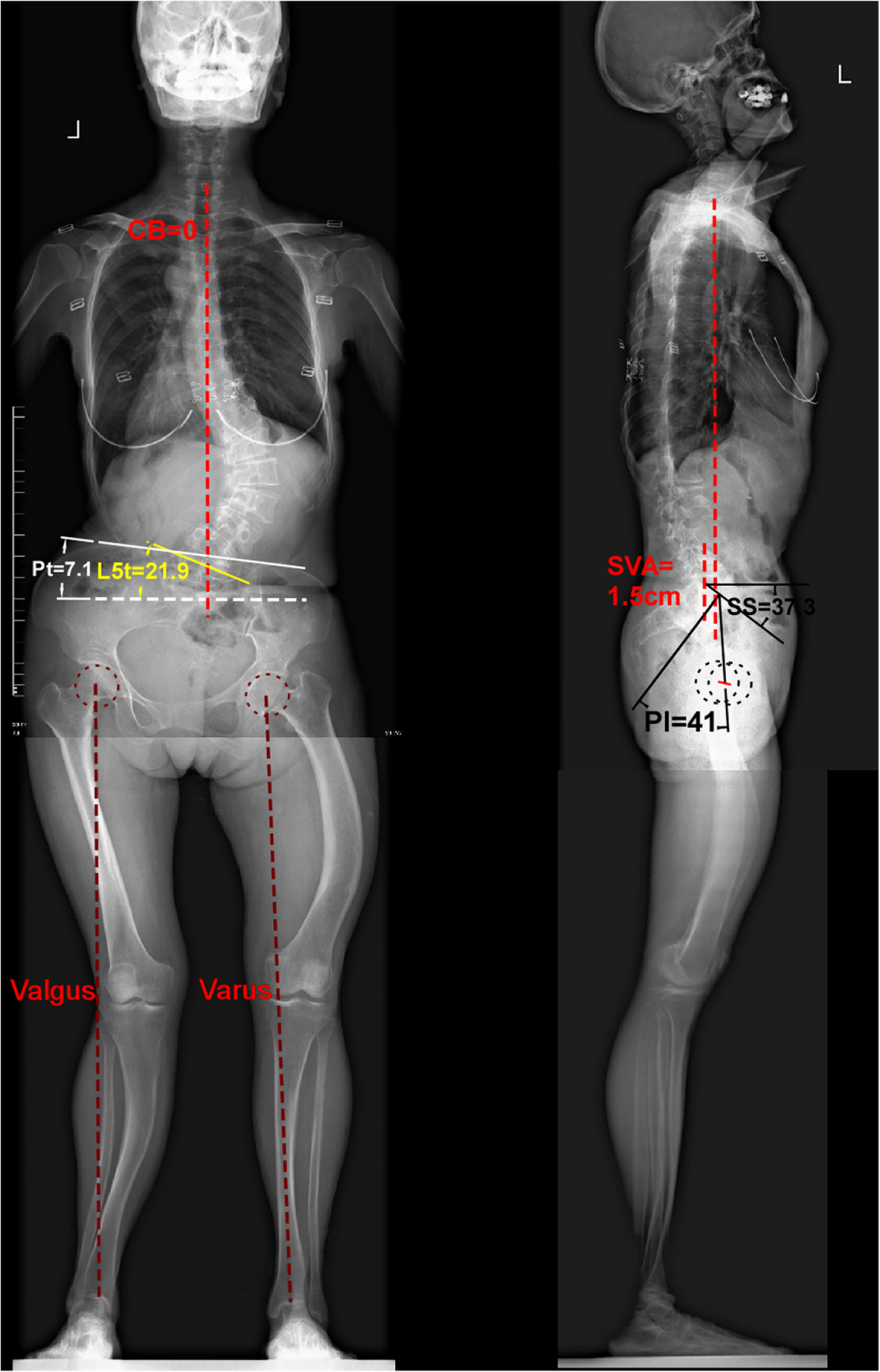
Standing whole length X-ray films before operation. The A-P film shows her main thoracolumbar (TL) curve Cobb is 72.5° (between T10-L3) while lumbosacral (LS) semi-curve Cobb (between L3-S1) is 44.9°. The length of left leg is 66.5 cm that is 2.5 cm longer than right leg (64.1 cm). The leg length discrepancy leads to pelvic obliquity with the pelvic tilt (Pt) angle is 7.1° (left higher than right). And L5 tile (L5t) angle is 21.9°. The coronal balance measured as distance between C7 centre point to CSVL is 0 at this time. The mechanical axis (brown dashed) shows the valgus leg in left side and the varus leg in right side. The bowed long bone deformities are shown in both legs. The lateral film (right side) shows her Pelvic incidence (PI) is 41°, sacral slope (SS) 37.3°, lumbar lordosis (LL) 46.5°, thoracic kyphosis (TK) 15.2° and sagittal vertical axis (SVA) 1.5 cm. Those parameters show her sagittal balance is good

Standing full-spine and full-lower limbs X-films are collected by the multi-purpose Digital R/F System (Sonial Vision Safire 17, Shimadzu Corporation). A-P film showed double structural curves (Fig. 1): the main thoracolumbar (TL) curve with Cobb angle from T10-L3 was 72.5° (43.1° in reduce position, Fig. S1), the lower semi-lumbosacral curve with Cobb angle from L3-S1 was 44.9°. C7-CSVL distance was 0. The lower limbs films (Fig. 1) showed clearly her bowed femur and tibia deformities. And the left leg was valgus deformity with the right leg was varus deformity that often been called as windswept deformity. Lumbar CT and MRI showed the L5-S1 segment is good (Fig. S2).

**Fig. 2:**
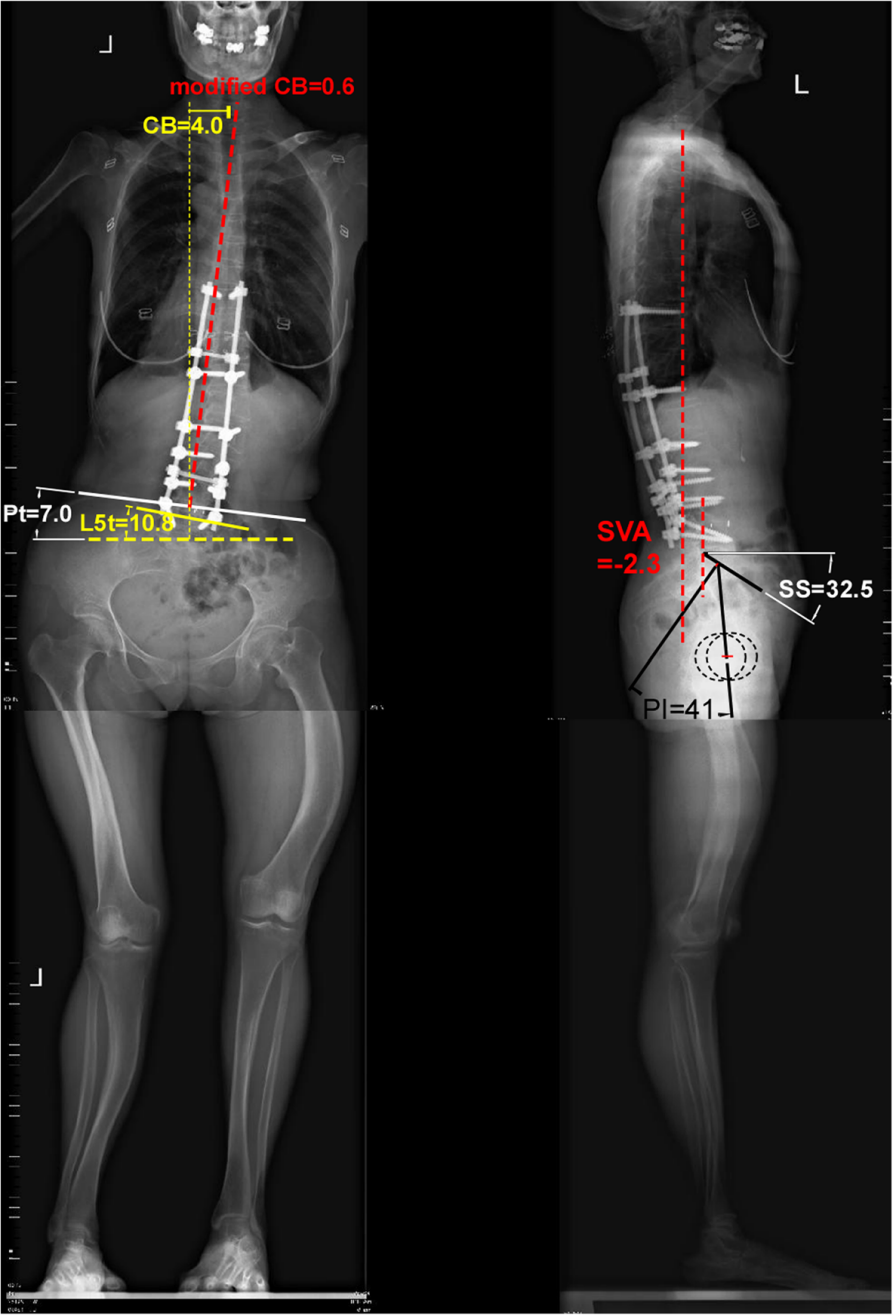
Standing whole length X-ray films at 2 years after operation. The A-P film (left side) shows her main thoracolumbar (TL) Cobb is 19.5° while lumbosacral (LS) Cobb is 16.7°. Pelvic tilt (Pt) angle is 7.0° while L5 tile (L5t) angle is 10.8°. Coronal balance (CB) means the distance between C7 centre point to CSVL line (yellow plumb dashed). Its value is 4.0 cm that means radiographic coronal imbalance. However, when we draw the modified CSVL (red dashed) based on the connect line of bilateral iliac highest point (white solid line), measure the distance between C7 centre point to modified CSVL and find the modified coronal balance (modified CB) is 0.6 cm (< 2 cm) which means coronal balance. The lateral film (right side) shows her sagittal balance is well maintained at this time

**Fig. 3:**
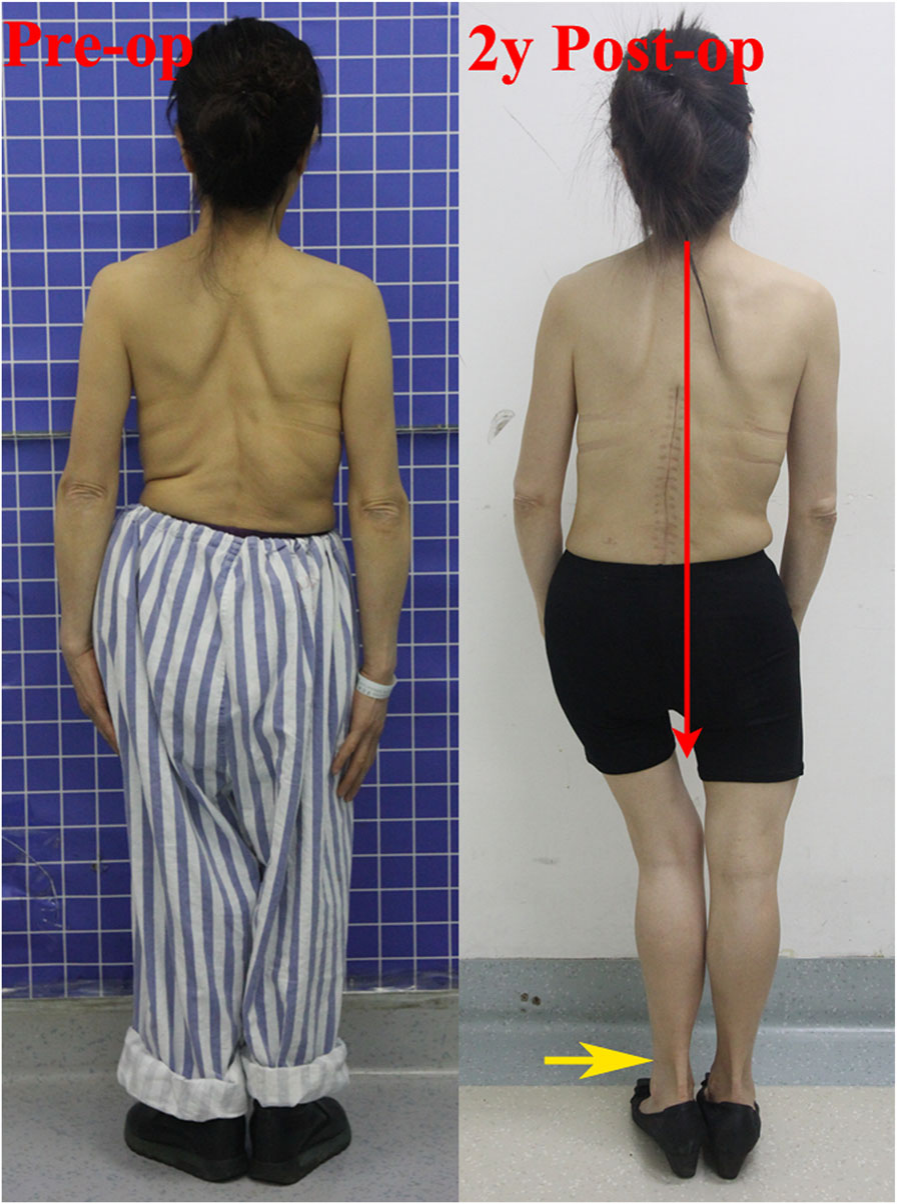
Preoperative (left) and 2 years postoperative appearance (right). Interesting phenomenon is the patient spontaneously moves her left foot insider (yellow arrow) to reduce the practical leg length discrepancy and then get better trunk balance (red line shows the line of gravity) after operation

In March 2017, a posterior correction (multilevel SPO) and fusion was performed at T9-L5. After surgery, her painful was great relieved (VAS = 3). The main TL curve was corrected to 21.0°, the coronal balance to 2.0 cm and the SVA to − 1.0 cm after surgery. She was asked to wear a thoracolumbar brace strictly for 3 months due to the relatively lower internal fixation density. At the 2 years follow-up, she had almost no pain (VAS = 2) and been very satisfied to the surgery. CT scans showed the bone graft has already fused (Fig. S3). No screw loose or hardware failure happened. The main scoliosis correction was well maintained (TL Cobb = 19.5°), but the posterior coronal imbalance seems appeared with standard C7-CSVL distance equal to 4.0 cm (Fig. 2). In fact when we artificially eliminate the pelvic tilt angle, the true spine coronal balance was very good (modified CB line in Fig. 2). Given the coronal imbalance was asymptomatic and can be improved by standing posture (Fig. 3). We advised her shoe lifts (2.5 cm high, on right foot) and asked her keeping follow-up.

## Discussion

Rickets is still a major public health issue, especially in the developing countries and in some ethnic groups living in more affluent countries [10]. The children usually perform bow-legs or knock-knees depending on the age of the child and the weight-bearing patterns in the limbs. Windswept deformity is a special type of lower limbs deformities with one valgus leg and the other varus one [7, 8]. All these types of lower limbs deformity may result in the LLD and asymmetric posture. Raczkowski et al. [5] analyzed the 5–17 years old children and indicated a slight primary LLD can cause functional scoliosis during growth and may require equalization treatment to prevent postural deterioration. And in another study by Ploumis et al. [11], they found the patients with LLD > 1 cm showed always pelvic obliquity and scoliotic curves. In our patient, her LLD was 2.5 cm so it’s easy to explain the occurring and developing of her scoliosis.

The classic treatment for rickets includes the 1,25(OH)_2_ vitamin D and calcium supplementation in the childhood. Once the lower limbs deformity appears, serial casting or surgeries are recommending. For the windswept deformity, both the earlier start hemiepiphysiodesis (i.e. eight-plate, Orthofix) and lower limb orthopedic surgeries (using Ilizarov or Taylor spatial frames) are useful to correct the deformities and LLD then preventing the subsequent scoliosis [8]. Effective early intervention is conventional nowadays, that is main reason why the case with windswept deformity associated with severe degenerative scoliosis is rarely reported in literature. Unfortunately, our patient has not received routine early treatment for her rickets or windswept deformity in her past decades. We also discussed this patient with some joint surgeons in our department, and they found the major problem is poor lines of force in lower limbs, rather than the hip or knee osteoarthritis. If we want to correct this patient’s LLD and pelvic obliquity, we need to try legs force lines correction by osteotomy of paramorphia femur and tibia preferentially. In fact, this is very difficult for patients to understand and accept, especially for such patient who has no lower extremities symptoms. For this patient, we suggested her to receive lower limb correction in advance of spine surgery, but she refused. And then we made the decision to do the spine surgery for her after a comprehensive communication.

Posterior long fusion is not in dispute in treating such severe DS patient. But the problem is which level should be chosen for lower instrumented vertebra (LIV). The basic rule in scoliosis correction indicates that the fusion is better to contain all the structural curved segments. However, this rule is hard to achieve in our patient. Because she performs double structural curves in lumbar (main TL curve and semi-lumbosacral curve). The pelvic obliquity and LLD actually are taking parts in the lumbosacral curve (Fig. 1). Fusion all structural curved segments, means we must take the lower limb orthopedic surgery into account. Excluding this option, the alternative LIV in fact can be chosen among L5, S1 or iliac, routinely.

Long fusion stopping at L5 can preserve L5-Sacrum motion segment that will reduce the concentration of forces applied to fixation segments and possibly decrease the incidence of pseudarthrosis [12]. But it also may accelerate L5-S1 disc degeneration, leading to pain and possibly a second surgery [13]. Stopping long fusion at sacrum gives more correction for the coronal deformity and resolves the L5-S1 disc problem simultaneously. However, it is often accompanies by much higher risk of instrumented failure and pseudarthrosis than LIV = L5 group [12, 14]. So in recent years, fusion at sacrum is gradually replaced by iliac fixation techniques [15]. Reasons surgeons favor fusing to the sacrum or iliac instead of stopping at L5 include (1) previous decompression at L5; (2) spondylolysis or listhesis at L5; (3) preoperative advanced disc degeneration at L5-S1 [16]. None of this performed in our patient, and that was the reason we chose LIV at L5 for her. Finally, the patient’s pain almost completely relieves with a radiographic satisfied deformity correction and bony fusion. Although, a new issue accompanying: postoperative coronal imbalance.

Coronal imbalance is one of common complications in DS. Bao et al. [17] reported the incidence of the coronal imbalance after surgery was up to 30% in these patients, even with the pelvic fixation. The L5 horizontality has been emphasized as the foundation of coronal spinal balance. Both the pre- and post-operative L5 tilt angle was large in our patient. It may be main reason for her coronal imbalance after surgery. Another important reason still points to the uncorrected structural pelvic obliquity and LLD (Fig. 2). In pursuit of ideal global trunk balance, pelvic fixation is usually recommended. Some scholars even suggest using sequential correction technique with multi-rod fixation system [17, 18]. Undoubtedly, with the pelvic fixation in the primary surgery, this patient should have much better coronal balance immediately after surgery. However, we are not sure whether the walking postural change or abnormal gait will come alone with pelvic fixation in such patient with structural LLD and pelvic obliquity. The long term surgical effect and patients’ quality of life of pelvic fixation in structural LLD patient is still unclear in current literatures.

The revision surgery (extend to pelvic fixation) is no need to our patient due to her present radiographic coronal imbalance is actual asymptomatic up to now. And we find another very interesting phenomenon is that the patient can self-adjust her coronal balance via moving her left foot close to right one in the standing position (which reducing the LLD relatively) (Fig. 3). So we recommend the shoe lifts to correct her LLD and then improve her trunk balance.

The limitation of this case is the follow-up is relatively short. In future, long-term follow-up is required to observe the further change of her lower limbs deformity and the risk of late deterioration due to coronal unbalance. Although the patient’s fixation is stable and bone graft is fused now, longer observation should be necessary to check the risk of mechanical complication (like rod broken, screw pull out, or PJK), especially in the upper instrumented level where the density of fixation is relatively low.

## Conclusions

Untreated windswept lower limbs deformity onset in childhood will easily lead to LLD then result in pelvic obliquity and progressive lumbar scoliosis. The Early intervention for primary disease (e.g. rickets) and the lower extremity orthopedic surgery before puberty should be emphasized. The sequence of spine or lower extremity surgery remains an open question in these patients, a relative reliable advise is treating the prominent symptom site firstly (such as this patient, chose spine correction prior to lower limbs). It’s very important whether we do the spine first or lower limbs surgery first, we must take another part into account when we design operation plan. The pure spinal correction and fusion surgery, in spite of lower limbs deformity, can achieve good relieve of back pain symptom, however, may accompany by the complication of coronal imbalance due to the unimproved pelvic obliquity and LLD. Longer follow-up is still required to observe the long-term outcome of this patient’s coronal imbalance. Whether pelvic fixation is better in such patients combined with lumbar deformity and structural LLD, this issue is worthy of further studies.

## Supplementary information

**Additional file 1: Figure S1.** Preoperative lateral bending whole spine X films: The reduce bending Cobb angle of main thoracolumbar curve is 43.1°, while increase one is 71°. The reduce bending Cobb angle of lumbosacral curve is 28.3° while increase one is 40.5°.

**Additional file 2: Figure S2.** Preoperative lumbar MRI shows L5-S1 disc is well.

**Additional file 3: Figure S3.** Two years postoperative CT sagittal scans shows satisfied fusion has been achieved.

## Data Availability

Data will be available upon request to the corresponding author.

## Abbreviations

LLD: Leg length discrepancy
DS: Degenerative scoliosis
TL: Thoracolumbar
CB: Coronal balance
