# Supplementary figures and images for: A case report of severe degenerative lumbar scoliosis associated with windswept lower limb deformity
Source: BMC Surg. 2020 Sep 3;20:195. doi: 10.1186/s12893-020-00857-x (PMC7470442; doi:10.1186/s12893-020-00857-x)

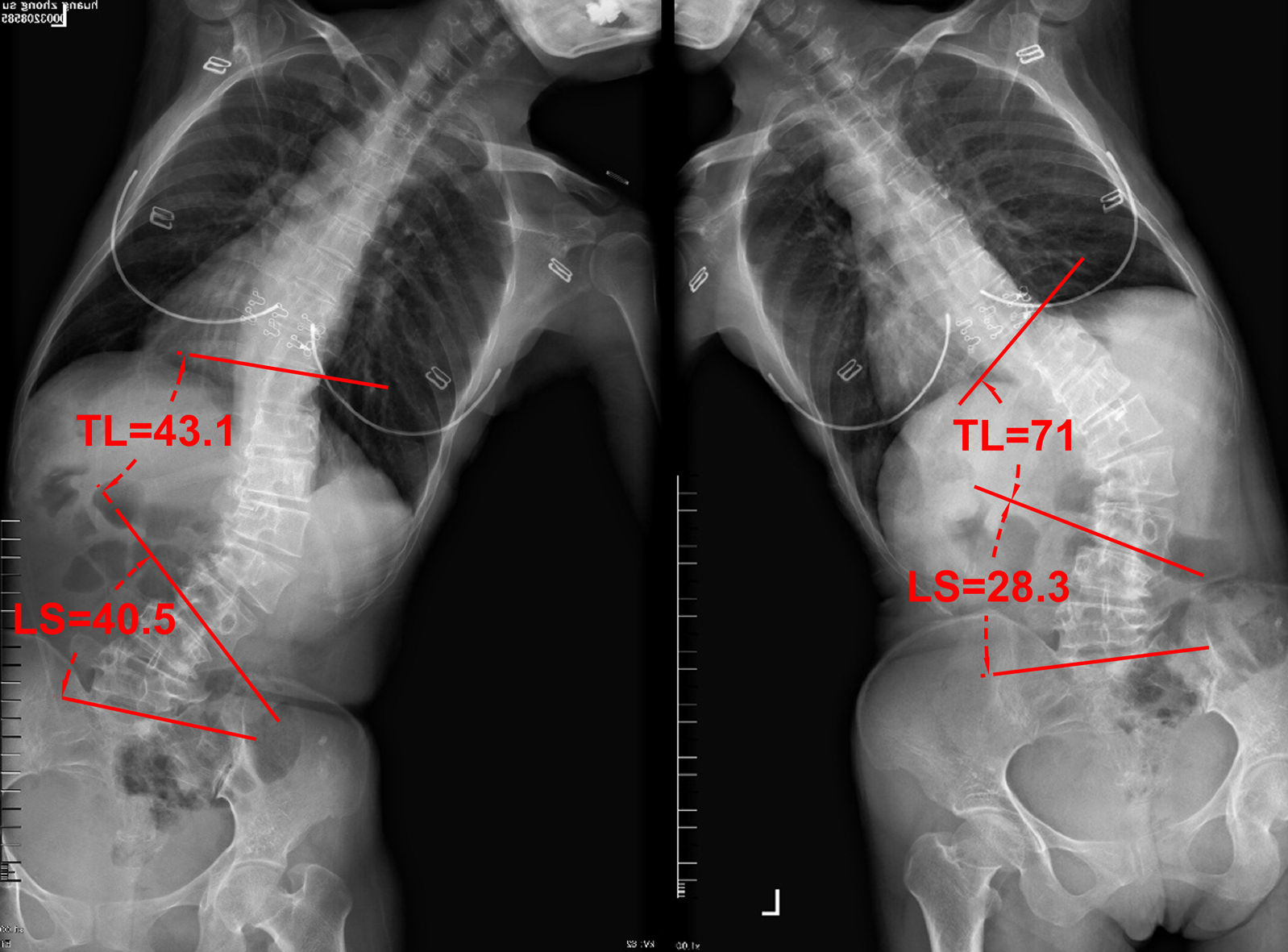

Supplement: Supplementary file 1 — Additional file 1: Figure S1. Preoperative lateral bending whole spine X films: The reduce bending Cobb angle of main thoracolumbar curve is 43.1°, while increase one is 71°. The reduce bending Cobb angle of lumbosacral curve is 28.3° while increase one is 40.5°. [file 12893_2020_857_MOESM1_ESM.tif]

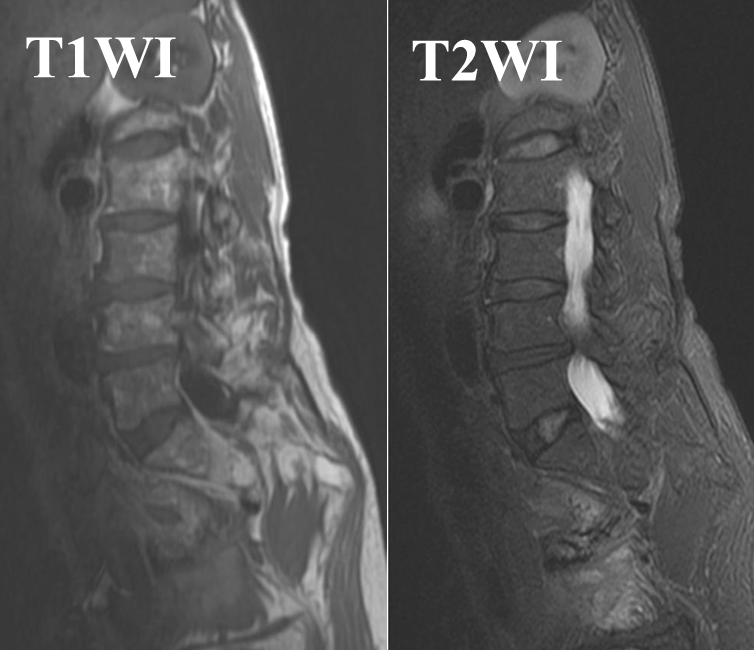

Supplement: Supplementary file 2 — Additional file 2: Figure S2. Preoperative lumbar MRI shows L5-S1 disc is well. [file 12893_2020_857_MOESM2_ESM.tif]

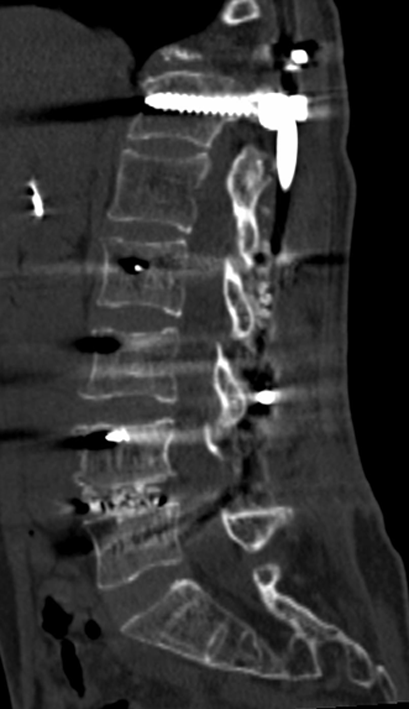

Supplement: Supplementary file 3 — Additional file 3: Figure S3. Two years postoperative CT sagittal scans shows satisfied fusion has been achieved. [file 12893_2020_857_MOESM3_ESM.tif]
